# Overweight and obesity profiles in Niger Delta Region, Nigeria

**DOI:** 10.4102/phcfm.v6i1.542

**Published:** 2014-01-28

**Authors:** Alphonsus U. Idung, Festus Abasiubong, Sunday B. Udoh, Uwemedimbuk S. Ekanem

**Affiliations:** 1 Faculty of Clinical Sciences, Department of Family Medicine, College of Health Sciences, University of Uyo, Nigeria; 2 Faculty of Clinical Sciences, Department of Psychiatry, College of Health Sciences, University of Uyo, Nigeria; 3 Department of Community Health, Faculty of Clinical Sciences, College of Health Sciences, University of Uyo, Nigeria

## Abstract

**Introduction:**

Overweight and obesity are global public health problems because of their effect on individuals, families and communities. The objective of this study was to describe the profile of overweight and obesity amongst adult outpatients in Uyo in the Niger Delta region of Nigeria.

**Method:**

This was a cross-sectional descriptive study done between October 2011 and March 2012. Using a systematic sampling technique, 584 subjects aged 18–65 years were recruited; data were collected with a structured questionnaire. Subjects were measured for height, weight, waist and hip circumferences. Body mass index (BMI) and waist–hip ratio (WHR) were calculated. Subjects with a BMI of 25.0 kg/m^2^ – 29.9 kg/m^2^ were regarded as being overweight whilst a BMI of > 30.0 kg/m^2^ was regarded as obese. Subjects with a WHR of > 0.90 for men or > 0.85 for women were regarded as having abnormal WHR.

**Results:**

Of the 584 subjects, 196 (36.6%) were men and 388 (66.4%) women. The mean age for men and women was 43.3 ± 17.8 years and 50.2 ± 13.6 years, respectively. The prevalence of overweight amongst men was 39.8% versus 31.7% for women; obesity in men was 28.0% versus 52.0% in women. Overweight and obesity were more prevalent in subjects aged 25–54 years and amongst married subjects. There was a significant relationship between obesity and television viewing (*p* = 0.003). Hypertension (*p* = 0.008) and osteoarthropathies (*p* = 0.043) were more prevalent amongst the obese than the non-obese subjects.

**Conclusion:**

Overweight and obesity are now common in our environment. There is therefore a need for more public education about the health consequences of big body size.

## Abstrait

**Profils de surpoids et d'obésité dans la région du delta du Niger au Nigeria**

**Introduction:**

Le surpoids et l'obésité sont des problèmes mondiaux de santé publique du fait de leur effet sur les individus, les familles et les communautés. L'objectif de cette étude était de décrire le profil de surpoids et d'obésité parmi les patients ambulatoires adultes à Uyo, dans la région du delta du Niger, au Nigeria.

**Méthode:**

Il s'agissait d'une étude descriptive transversale effectuée entre octobre 2011 et mars 2012. 584 sujets de 18-65 ans ont été recrutés selon une technique d’échantillonnage systématique; les données ont été obtenues à l'aide d'un questionnaire structuré. La taille, le poids, les tours de taille et de hanches ont été mesurés. L'Indice de masse corporelle (IMC) et le Rapport taille-hanches (RTH) ont été calculés. Les sujets présentant un IMC compris entre 25.0 et 29.9 kg/m^2^ étaient considérés comme étant en surpoids, alors qu'un IMC > 30.0 kg/m^2^ était considéré comme de l'obésité. Les sujets présentant un RTH > 0.90 pour les hommes ou > 0.85 pour les femmes étaient considérés comme présentant un RTH anormal.

**Résultats:**

Parmi les 584 sujets, 196 (36.6%) étaient des hommes et 388 (66.4%) des femmes. L’âge moyen pour les hommes et les femmes était respectivement de 43.3 ± 17.8 ans et 50.2 ± 13.6 an. La prévalence de surpoids parmi les hommes était de 39.8% contre 31.7% pour les femmes; l'obésité chez les hommes était de 28.0% contre 52.0% chez les femmes. Le surpoids et l'obésité étaient plus répandus chez les sujets âgés entre 25 et 54 ans et parmi les sujets mariés. On a pu observer une relation significative entre l'obésité et le fait de regarder la télévision (*p* = 0.003). L'hypertension (*p* = 0.008) et les ostéo-arthropathies (*p* = 0.043) étaient plus répandues parmi les sujets obèses que chez les non-obèses.

**Conclusion:**

Le surpoids et l'obésité sont maintenant courants dans notre environnement. Il est donc nécessaire d’éduquer davantage le public à propos des conséquences sur la santé d'une grande taille corporelle.

## Introduction

Overweight and obesity are a global public health problem because of their effect on individuals, families and communities. It is estimated that about one billion people are overweight and more than 300 million obese worldwide.^1^

Overweight and obesity are reported to account for 44% of the global burden of diabetes mellitus, 23% of ischaemic heart disease and 7% – 41% of some malignancies.^1^ It is estimated that of the projected 64 million deaths worldwide in 2015, 41 million (64%) will result from non-communicable diseases (NCDs), of which obesity is a major contributor.^2^

The World Health Organization (WHO) defines ‘overweight’ as a body mass index of 25.0 kg/m^2^ – 29.9 kg/m^2^ and ‘obesity’ as a body mass index of > 30.0 kg/m^2^.^2^ The body mass index (BMI) is usually calculated by dividing the weight in kilograms by the square of the height in metres (BMI = Wt [kg] / Ht^2^ [m]).^3^ Its flaw lies in the fact that it may misclassify a very muscular person as obese and it may also overestimate fatness in hypertrophied athletes or underestimate fatness in the elderly.^4^ Body mass index also cannot estimate intra-abdominal fat which is an independent predictor of health risk.^5^

The waist-hip ratio (WHR) as a measure of obesity has been judged to be very valuable as it provides an index of the proportion of intra-abdominal fat.^4, 5^ The normal waist circumference for women is 88 cm or less, whilst the normal waist circumference for men is 102 cm or less. Average waist-hip ratio values for men and women are about 0.93 (range 0.75–1.10) and 0.83 (range 0.70–1.0),^4, 5^ respectively. Some have, however, combined BMI with the measure of waist circumference in order to predict disease risk in patients.^6^

The aetiology of obesity is poorly understood, but genetic, environmental, socioeconomic and viral factors have been implicated.^7, 8^ Other factors include lack of sufficient physical exercise arising from unplanned urbanisation, the lure of technology and safety fears. It has also been documented that adults who watch four or more hours of television each day are four times more likely to be overweight or obese than those who spend less time in this pursuit.^8^ Overweight and obesity are on the increase in Africa and might assume an epidemic dimension in the near future.^9^ The reported prevalence of overweight and obesity amongst urban dwellers in Africa is 35% and it is estimated that by 2025, 75% of the obese people worldwide will reside in the developing world.^1, 9^

The reported prevalence of overweight and obesity amongst urban dwellers in Jos, North Central Nigeria is 21.4%, made up of 19.4% males and 23.5% females, of whom 17.2% were overweight whilst 4.2% were obese.^10^ Another study from Lagos, South-West Nigeria, reported the prevalence of obesity amongst urban dwellers to be 6.9%.^11^

Published reports on the prevalence of overweight and obesity in Uyo, a community in the Niger Delta Region, South-South Nigeria are, however, scarce in the scientific literature. This study is therefore aimed at describing the occurrence of overweight and obesity amongst family medicine outpatient clinic attendees in Uyo.

It is hoped that the findings will add to the pool of available knowledge, for the purpose of increasing awareness on lifestyle issues in the region which could help prevent the epidemic of overweight and obesity with its attendant health consequences.

## Research methods and design

### Location of the study

This study was carried out at the family medicine outpatient clinic of the University of Uyo Teaching Hospital (UUTH). UUTH is located on the outskirts of Uyo, the capital of Akwa Ibom State in Nigeria. Nigeria is divided into six geopolitical zones as follows: North-East, North-West, North-Central, South-East, South-West and South-South. Uyo is located in the South-South geopolitical zone, which is often referred to as the Niger Delta Region of Nigeria. It is one of the major oil-producing areas in the region. UUTH is the only tertiary and referral health institution in the State and its environs, serving a population of approximately 3.9 million people.

### Subjects

The study was carried out amongst outpatients attending the family medicine clinic for various medical problems. They were recruited using a systematic random-sampling method with a sampling interval of six. Each recruited subject was given information regarding the research objectives after written informed consent was obtained. Numbers ranging from one to six were assigned to the first six subjects who met the inclusion criteria. The first respondent was chosen by simply balloting one of the numbers from a basket containing the assigned numbers. Subsequently, every sixth subject was recruited into the study. Where, however, a subject was judged to be too ill at the point of recruitment into the study, that subject was dropped and the next subject who met the inclusion criteria was recruited. Other exclusions included pregnant women as well as subjects who were below the age of 18 or above 65 years of age.

### Methods

This study, with a sample of 584 consenting male and female subjects, took place between October 2011 and March 2012. Weight was measured in kilograms to the nearest 0.5 kg using a Hanna-calibrated bathroom scale, model BR9011. Each subject was weighed wearing light clothing without shoes or stockings. The height of the subjects was measured using an improvised wooden stadiometer mounted on a vertical wall with the respondent standing erect against the wall on a horizontal floor without shoes. The head was placed so as to ensure that the external auditory meatus and the angle of the eye were on a horizontal line. The height was measured in metres to the nearest 0.1 cm. BMI was calculated as the weight (kg) / (height [m])^2^ (i.e. kg/m^2^).

A non-stretch tape was used to measure the body circumference. The smallest circumference between the xiphisternum and the umbilicus on expiration was taken as being the waist circumference. Measurements were taken to the nearest 0.1 cm after normal expiration with the subject in an upright position. Hip circumference was measured to the nearest 0.1 cm at the maximum posterior protuberance of the buttocks whilst the subject was standing upright with feet together.

Moreover, each of the subjects was assessed using an interviewer-administered structured questionnaire containing such information as age, marital status, highest level of education attained, income (in Nigerian Naira denomination) using the approved wage structure in the Nigerian public service.^12^ Other information contained in the questionnaire included the occupational status of the respondents, which was classified as non-skilled (including labourers, pensioners and students), skilled (including tailors and teachers without a University degree) and professional (including lawyers, teachers with University degree and soldiers). The questionnaire also elicited information with regard to the average number of hours spent each day watching television and the number of days spent each week engaging in any form of structured physical exercise such as cycling, rope-skipping, trekking and/or hiking and other forms of aerobic physical exercise.

### Data analysis

The statistical analysis was done using SPSS Statistics version 17.0 (IBM Corporation), distribution and cross-tabulation data were generated, the *t*-test was used to compare means and Chi-square was used to compare proportions. A *p*-value of 0.05 was used to determine the level of statistical significance.

### Ethical considerations

Ethical approval for the study was obtained from the UUTH ethical committee (UUTH/AD/S/96/VOL.1X/969). A pretest of the research questionnaire was performed in order to determine its applicability, experience and logistical problems.

## Results

Of the 584 subjects recruited into the study, 36.6% (*n* = 196) were men, whilst 66.4% (*n* = 388) were females.

Table 1 shows the sociodemographic and clinical characteristics of the subjects. There was a statistically-significant difference between the mean age of women (50.2 [SD = 3.6]) and men (43.3 [SD = 17.8]) (*p* = 0.000). Subjects between the ages of 25 and 54 were more obese (*p* = 0.000). Overweight and obesity was more prevalent amongst the married respondents in this study (*p* = 0.002). Women had a mean BMI of 30.7 kg/m^2^ (SD = 5.7) versus 27.6 kg/m^2^ (SD = 4.5) for men (*p* = 0.000). Women had a mean waist circumference of 95.3 cm (SD = 12.4) versus 91.2 cm (SD = 11.2) for men (*p* = 0.000). The mean hip circumference for women was 109.4 cm (SD = 14.2), whilst the mean hip circumference for men was 103.3 cm (SD = 10.4) (*p* = 0.000). Approximately 65.0% of the women had an abnormal WHR compared with 37.8% of the men.

**TABLE 1:** Characteristics of the study population.

Variables	Subjects	

	Female (F) (*n* = 388 [%])	Male (M) (*n* = 196 [%])
**Socio demographic age in years**		
18–24	9 (2.3)	12 (6.1)
25–34	120 (30.9)	38 (19.4)
35–44	118 (30.4)	58 (29.6)
45–54	94 (24.2)	56 (28.6)
55–64	42 (10.8)	20 (10.2)
65	5 (1.3)	12 (6.1)
**Marital status**		
Single	64 (16.5)	45 (23.0)
Married	281 (72.4)	145 (74.0)
Divorced and/or separated	8 (2.1)	2 (1.0)
Widow	35 (9.0)	4 (2.0)
**Educational level**		
No formal education	8 (2.1)	1 (0.5)
Primary school	54 (13.9)	25 (12.8)
Secondary school	92 (23.7)	51 (26.0)
Post-Secondary school	234 (60.3)	119 (60.7)
**Income**		
Low	217 (56.0)	96 (49.0)
Middle	108 (27.8)	65 (33.2)
High	63 (16.2)	35 (17.8)
**Occupation**		
Non-skilled	75 (19.3)	28 (14.3)
Skilled	115 (29.7)	69 (35.2)
Professional	198 (51.0)	99 (50.5)
**Place of residence**		
Urban	244 (62.9)	123 (62.8)
Rural	144 (37.1)	73 (37.2)
**Body Mass Index (kg/m^2^)**		
< 18.5	-	-
18.5–24.9	63 (16.2)	63 (32.1)
25.0–29.9	123 (31.7)	78 (39.8)
30.0–34.9	108 (27.8)	44 (22.4)
35.0–39.9	69 (17.8)	9 (4.6)
≥ 40.0	25 (6.4)	2 (1.0)
**Waist circumference**		
≤88 cm (F)	84 (21.6)	-
≤102 cm (M)	-	171 (87.2)
≥88 cm (F)	304 (78.4)	-
≥102 cm (M)	-	25 (12.8)
**Quartiles of waist-–hip-ratio**		
≤0.84	136 (35.0)	57 (29.1)
0.85–0.89	142 (36.6)	65 (33.1)
> 0.93	110 (28.4)	74 (37.8)

Table 2 shows the lifestyle characteristics of the respondents. The number of hours spent watching television each day was a significant contributor to obesity in this study (*p* = 0.003). Conversely, the number of days spent doing structured physical exercise did not contribute significantly to obesity in this study (*p* = 0.72).

**TABLE 2:** Lifestyle characteristics of the subjects.

Lifestyle characteristics	Subjects		

	Non-obese (*n* = 327 [%])	Obese (*n* = 257 [%])	*p*-value
**How many hours do you spend to watch television each day?**			
< 4 hours	273 (83.5)	189 (73.5)	0.003*
≥4 hours	54 (16.5)	68 (26.5)	-
**How many days do you engage in structured physical exercise per week?**			
None	201 (61.5)	150 (58.4)	-
1–4 days (each episode lasting up to 30 minutes)	94 (28.7)	78 (30.4)	0.72
> 5 days (each episode lasting up to 30 minutes)	32 (9.8)	29 (11.2)	-

* Statistically significant.

Table 3 shows the prevalence of medical conditions amongst the respondents in this study. There was a statistically-significant difference (*p* = 0.008) between the presence of hypertension and the presence of osteoarthropathies (*p* = 0.043) when comparing the obese versus the non-obese subjects in this study.

**TABLE 3:** Prevalence of medical conditions of subjects.

Medical history	Subjects		

	Non-obese (*n*[%])	Obese (*n*[%])	*p*-value
Hypertension	104 (31.8)	110 (42.8)	0.008*
Diabetes mellitus	48 (14.7)	48 (18.7)	0.24
Osteoarthropathies	25 (7.6)	33 (12.8)	0.043*
Others (malaria, dyspepsia, enteric fever etc.)	24 (7.3)	23 (8.9)	0.58

* Statistically significant

## Discussion

Malnutrition, particularly undernutrition and starvation, have long been a major problem in Africa, particularly amongst the inhabitants of rural areas. Interestingly, however, in our study we found a high prevalence of obesity amongst our subjects (52.0% for the women versus 28.0% for the men). This figure is, however, still lower than the prevalence rates of 71.6% in female and 50.5% in male hypertensive patients in Nigeria.^13^

In another population-based study, obesity was reported amongst 21% of the male and 28% of the female respondents.^14^ The differences in the prevalence of obesity amongst different workers in Nigeria might be due to the differences in the study design as well as the study subjects, but it is nonetheless important to take note of the fact that obesity is no longer only a problem of the affluent or of those in developed countries.

The reasons for the increasing prevalence of obesity amongst the respondents in this study may be due to the fact that Uyo and its environs, which were predominantly an agrarian subsistent farming community less than a decade ago, are now changing into a modern metropolis because of Uyo's currents status as a State capital, coupled with rapid but unplanned urbanisation as well as a possible change from local dietary patterns.

The high prevalence of overweight women (31.7%) and men (39.8%) in this study suggests that the obesity rate will continue to rise unless drastic action is taken. The prevalence of obesity was higher in women than men, a finding that is consistent with other studies in Nigeria and various parts of Africa._10, 13, 14, 15_

In Ghana, obesity is reported to be six times more common in women than in men and in Morocco, four times more common.^16^ Amongst Africans, including Nigerians, being overweight or obese is thought to reflect affluence and happiness, a factor that may seriously militate against any effort toward managing the problem. Married women were more obese than others in this study, a finding that is also consistent with reports from other studies.^17, 18^ The reason for this is that being overweight or obese is thought to reflect a husband's ability to take care of his wife and family – a serious misconception that needs to be addressed properly amongst those who are vulnerable.

Although it has been shown to be a possible contributor to overweight and obesity in this study, the role of television viewing, a largely sedentary activity, as a factor in the aetiology of overweight and obesity should be interpreted with caution.^8^ The reason for this is that other factors, such as the environment and genetics, must not be overlooked. Further studies in this regard are hereby advocated.

Finding from this study also show a high prevalence of chronic medical conditions amongst the respondents, which is consistent with reports from other studies.^13, 14, 19, 20^

The health consequences of obesity are real and serious. In addition, the economic burden in both the private and public sector is enormous, because the cost of treatment of obesity-related diseases are borne not only by the obese and their families, but also by all tax payers or contributors to the Nigerian National Health Insurance Scheme.

## Limitations

One limitation of this study is that the data used is cross-sectional – it would have been more appropriate to follow up with individuals over time so as to ascertain the changes in their BMI over time as well as the presence of other risk factors. Moreover, the population used in this study does not represent the entire adult population in the Niger Delta Region of Nigeria, as it is derived only from patients attending an outpatient clinic.

## Conclusion

The findings of this study have shown that overweight and obesity are prevalent amongst the outpatients seen in our hospital.

Considering the chronic nature of most disease associated with obesity as well as the enormous cost of treatment, the prospect of taking on a larger disease burden is alarming for the already poorly-funded and ill-equipped healthcare delivery system in the Niger-Delta Region of Nigeria, which is still grappling with the scourge of HIV. The scientific community is therefore encouraged to join in efforts to create more responsible solutions for the obesity epidemics at the societal level.

In view of the alarming presence of overweight and obesity amongst our respondents in particular and Nigerians in general, we recommend that citizens be encouraged to adopt a healthier lifestyle through good public sensitisation and education. Physicians and caregivers need some more training on motivational interview techniques in order to become more effective in motivating their overweight and obese patients to adopt healthier lifestyle. There is also a need to establish acceptable BMI cut-offs for overweight and obesity for Nigerians.

Family physicians should interact more with local health educators in order to promote collaboration amongst care providers and communities and to identify the most sustainable and community-friendly strategies to promote healthy weight.

There is also a need for interdisciplinary and collaborative research from family medicine, community health and urban planning so as to facilitate practice and policy changes that could foster broad population-level behavioural changes.
